# Performance of Microchannel Heat Sink Made of Silicon Material with the Two-Sided Wedge

**DOI:** 10.3390/ma15144740

**Published:** 2022-07-06

**Authors:** Aditya Vatsa, Tabish Alam, Md Irfanul Haque Siddiqui, Masood Ashraf Ali, Dan Dobrotă

**Affiliations:** 1 Department of Mechanical Engineering, Thapar Institute of Engineering and Technology, Patiala 147004, India; vatsadi25@gmail.com; 2 CSIR—Central Building Research Institute, Roorkee 247667, India; tabish.iitr@gmail.com; 3 Mechanical Engineering Department, King Saud University, Riyadh 11421, Saudi Arabia; msiddiqui2.c@ksu.edu.sa; 4 Department of Industrial Engineering, College of Engineering, Prince Sattam Bin Abdulaziz University, Al-Kharj 16273, Saudi Arabia; mas.ali@psau.edu.sa; 5 Faculty of Engineering, Department of Industrial Engineering and Management, Lucian Blaga University of Sibiu, 550024 Sibiu, Romania

**Keywords:** microchannel, heat sink, silicon material, wedge angles, Nusselt number

## Abstract

New designs of the microchannel with a two-sided wedge shape at the base were studied numerically. Five different wedge angles ranging from 3° to 15° were incorporated into the microchannel design. Simulation of this novel microchannel was carried out using Computational Fluid Dynamics (CFD). Three-dimensional models of the microchannel heat sink were created, discretized, and based on Navier–Stokes and energy equations; laminar numerical solutions were obtained for heat transfer and pressure drop. Flow characteristics of water as coolant in a microchannel were studied. It was observed that numerical results are in good agreement with experimental results. It was found that the Nusselt number and friction factor are significantly varied with the increase in Reynolds number. The Nusselt number varies in the following ranges of 5.963–8.521, 5.986–8.550, 6.009–8.568, 6.040–8.609, and 6.078–8.644 at 3°, 6°, 9°, 12°, and 15°, respectively. The microchannel with a wedge angle of 15° was found to be better in terms of Nusselt number and thermo-hydraulic performance. The enhancement in the Nusselt number is found as 1.017–1.036 for a wedge angle of 15°; however, friction factors do not show the perceptible values at distinct values of wedge angle. Moreover, the thermo-hydraulic performance parameters (THPP) were evaluated and found to be maximum in the range of 1.027–1.045 for a wedge angle of 15°. However, minimum THPP was found in the range of 1.005–1.0185 for a wedge angle of 3°.

## 1. Introduction

The improving and advancing technology has increased the human dependency on electronic devices. Currently, it is unimaginable to live without devices. The compact size of these devices has made them more common and popular. As with any other machinery, these devices also heat up, and thus thermal management is required in designing these devices for better long-run use. Many solutions were introduced for this purpose which had a remarkable effect on the reliability and operating life of the electronic devices [1,2,3].

One of the most common forms of thermal management currently used in technology, machinery, and natural systems is heat sinks. It is a passive heat exchanger that helps transfer the heat generated by a mechanical or electronic device to a fluid medium, usually liquid or air coolant. The heat is dissipated away from the device, and the appropriate device temperature is maintained. These are used in devices that are not self-sufficient to moderate their temperature, such as CPUs, GPUs, and RAM in computers; power transistors; lasers; LEDs that have high power transistors; and in aerospace technology.

Thermal management of electronic devices has grown rapidly in recent years since a new generation of high-performing dense chip packages that operate at high frequencies produces a very high heat flux on electronic devices. Modern electronic packages’ increasing power density and compactness stimulate the quest for efficient and small cooling components for high heat production processors. The key purpose of these components is to maintain the electronic packages within the working temperature range specified in the design. Unlike traditional heat sinks, which require a large surface area to enhance heat dissipation rates, microchannel heat sinks appear to be suitable due to their compact size and high performance. There has been a big interest in studying fluid flow and heat transmission in microchannels since Tuckerman and Pease’s [4] ground-breaking work in the early 1980s.

A microchannel heat sink is dependent on the use of minute diameter liquid-coolant channels. These tiny diameter passageways provide a wide surface for heat transmission between the chip and the coolant, hence enhancing the heat transfer rate [5]. The use of microchannel heat sinks in cooling electronic packages, on the other hand, imposes severe constraints on the package’s architecture. At a specific heat generation rate, the temperature rise, pressure drop, and coolant flow rate require modification of the microchannel heat sink to efficiently dissipate the heat.

Qu et al. [6,7] analyzed the flow properties of water through a trapezoidal silicon microchannel with hydraulic diameters in the range of 51 to 169 m. Because of the effect of microchannel surface roughness, their findings showed that the pressure gradient and flow friction in the microchannel are higher than those predicted by conventional laminar flow theory. In order to analyze the experimental results, they suggested a roughness–viscosity model. In microtubes with diameters of 19, 52, and 102 m, Yu et al. [8] investigated the fluid flow and heat transfer properties of dry nitrogen gas and water. In the microchannel, Pfahler [9] examined the apparent viscosity of isopropanol alcohol and silicon oil.

A complete evaluation of friction factor data in a microchannel with liquid flows was provided by Steinke and Kandlikar. They stated that while reporting total friction factor losses in microchannel, entry and exit losses must be taken into consideration. The conventional view is very well supported by the majority of the data that account for friction factor loss. They also proposed a new method for accounting for inlet and outlet exit losses when measuring pressure drop [10]. Toh et al. [11] studied the three-dimensional fluid flow and heat transport phenomena inside a heated microchannel. They used a finite-volume approach to solve the stable laminar flow and heat transfer equations. By comparing the anticipated local thermal resistances and friction factors to the available experimental data, the numerical technique was shown to be valid. They discovered that adding heat reduces frictional losses and viscosity, resulting in a rise in water temperature, especially at lower Reynolds numbers.

Tiselj et al. [12] analyzed the performance of axial conduction on heat transfer in a microchannel heat sink with a triangular microchannel numerically and experimentally. They noted that the temperatures of the bulk water and heated walls did not change linearly along the channel. Experiments were carried out in the work of Lee et al. [13] to investigate the validity of classical correlations based on standard-sized channels for forecasting thermal behavior in single-phase flow via rectangular microchannels. A numerical simulation was also performed, and the results were compared to the experimental data. They concluded that both fluid flow and heat transfer area in the developing regime should not be overlooked in the study.

Peng and Peterson [14,15] investigated the pressure drop and convective heat transfer of water flow in a rectangular microchannel. It was discovered that the cross-sectional aspect ratio had a significant impact on flow friction and convective heat transfer in both laminar and turbulent flows. Several studies recently focused on various features of microchannel geometry to improve heat transmission. Wu and Cheng [16] experimentally showed that, in addition to the different interfacial effects outlined above, the cross-sectional form of the channel could have a significant impact on fluid flow and heat transfer inside a noncircular microchannel. Furthermore, the abovementioned literature assessment revealed that there is relatively little research into the influence of geometrical factors on heat sink performance, particularly for pentagonal microchannels. As a result, the current research aimed to close the gap by investigating the effects of geometrical factors, Reynolds number, and heat flux on pressure drop and laminar convective heat transfer in a microchannel of various forms. The findings of this study on the influence of geometrical factors could be useful in a variety of industrial and natural processes where understanding heat transfer behavior is critical.

Zheng et al. [17] carried out a numerical simulation of the cone-column heat sink to study the performance in the Reynolds number range of 100 to 700. It was shown that the base temperature of this novel heat sink is lower than the base temperature of a similar circular microchannel heat sink. Rogie et al. [18] designed a new triangular finned microchannel and studied the pressure drop and heat transfer characteristics numerically. The study encompassed the effect of triangular fin pitch and transverse tube pitch in the following ranges of 2.50–10.0 mm and 9.0–21.0 mm, respectively. As a result, they developed the Fanning f-factor and Colburn j-factor correlations, which were able to predict the numerical results with the accuracy of 3.95% and 3.41%, respectively. Duan et al. [19] explored the laminar slip flow hydrodynamically. A semi-numerical model was developed to predict the pressure drop of microchannel plate-fin heat sinks. Rasangika et al. [20] exploited square wave-shaped vibration for performance improvement of a heat sink numerically. It was concluded that the square wave-shaped vibration of the heat sink has a significant improvement in thermal performance than the sinusoidal wave-shaped vibration of a heat sink. Kumar and Singh [21] carried out a numerical study on different-sized microchannels with micro inserts. It was reported that microchannels with inserts effectively enhanced the heat transfer performance by 1–9%; however, these micro inserts increased the pressure drop by 14.5%. Chen et al. [22] proposed a novel cross rib microchannel heat sink wherein it rotates the heat transfer fluid. The heat dissipation performance was evaluated numerically. The desirable results were found with the following percentage in cooling capability reported as 28.6% and 14.3% when compared with the rectangular and horizontal ribs heat sink and the cross-rib microchannel, respectively. Piasecka et al. [23] presented the experimental and numerical study on a heat sink with several asymmetrical heated mini-channels wherein heat transfer and flow characteristics were studied. The numerical results were validated with experimental results to ensure the good accuracy of various parameters measured. Wu et al. [24] proposed a liquid metal-based heat sink to explore the effect of the shape of cross-sectional of the microchannel, different working fluids, and various inlet velocities. It was reported that inlet velocity had a great influence on heat dissipation capability, which increased by about 74.35%. Jia et al. [25] exploited the oval-shaped micro pin fins to study heat transfer and fluid flow characteristics by encompassing the Reynolds number in the range of 157 to 668. The results showed that oval-shaped pin fins in the microchannel were able to suppress the relatively higher base temperature and provided a uniformly distributed temperature. Eneren et al. [26] presented a review paper wherein experimental studies on microchannels were summarized. In these studies, nanofluid was exploited as heat transfer fluids. Nanofluids as heat transfer fluids are gaining importance; however, they have adverse effects such as abrasion, erosion, corrosion, and amalgamation of nanoparticles.

Computational Fluid Dynamics (CFD) approaches are strong tools for simulating fluid flow and associated heat and mass transport by numerically solving mathematical equations that control these processes, making use of fast and ongoing advances in computers and computing techniques. Comprehensive, thorough analyses; conceptual studies of re-design and new designs; in-depth product research and development; and troubleshooting are all areas where CFD simulations are useful [27]. In comparison to analytical and experimental fluid dynamics, CFD is highly significant in simulations of micro-electromechanical-systems (MEMS) applications, notably in the design of effective microchannel heat sinks. When compared to experimental approaches, using CFD simulation models in manufacturing and design saves time and money [28,29]. CFD can also address a wide range of complicated issues that are unavailable to traditional analytical approaches. As a result, CFD enhances fundamental knowledge of fluid flow, mass, and heat transfer properties, which are critical in microchannel heat sink design and process management.

Based on the literature and keeping an eye on the gaps, this paper presents the effect of microchannel cross-sectional shape on heat transfer and friction characteristics. The base of the microchannel was designed in such a way as to increase the heat transfer area. The objective of this work was to analyze the effect of two-sided wedge angles of the base in a microchannel on heat transfer and friction characteristics using water as heat transfer fluid. The results were compared with the conventional microchannel to establish the enhancement in heat transfer capability.

## 2. Microchannel Heat Sink Computational Model

### 2.1. Computational Domain

**Figure 1:**
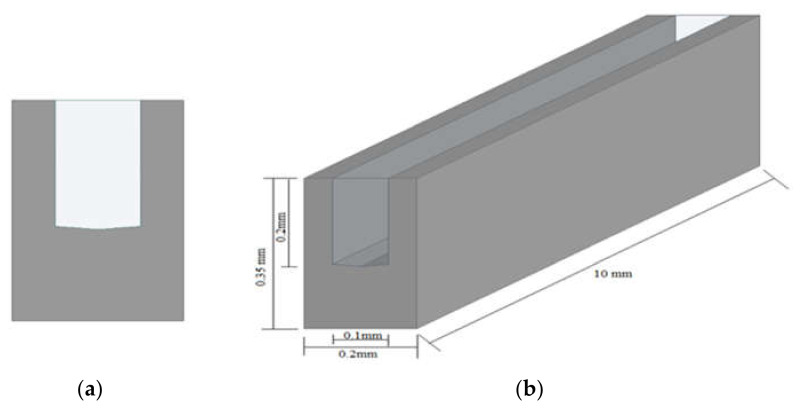
Dimensional computational domain of single microchannel: (**a**) cross-sectional view, (**b**) isometric view.

**Figure 2:**
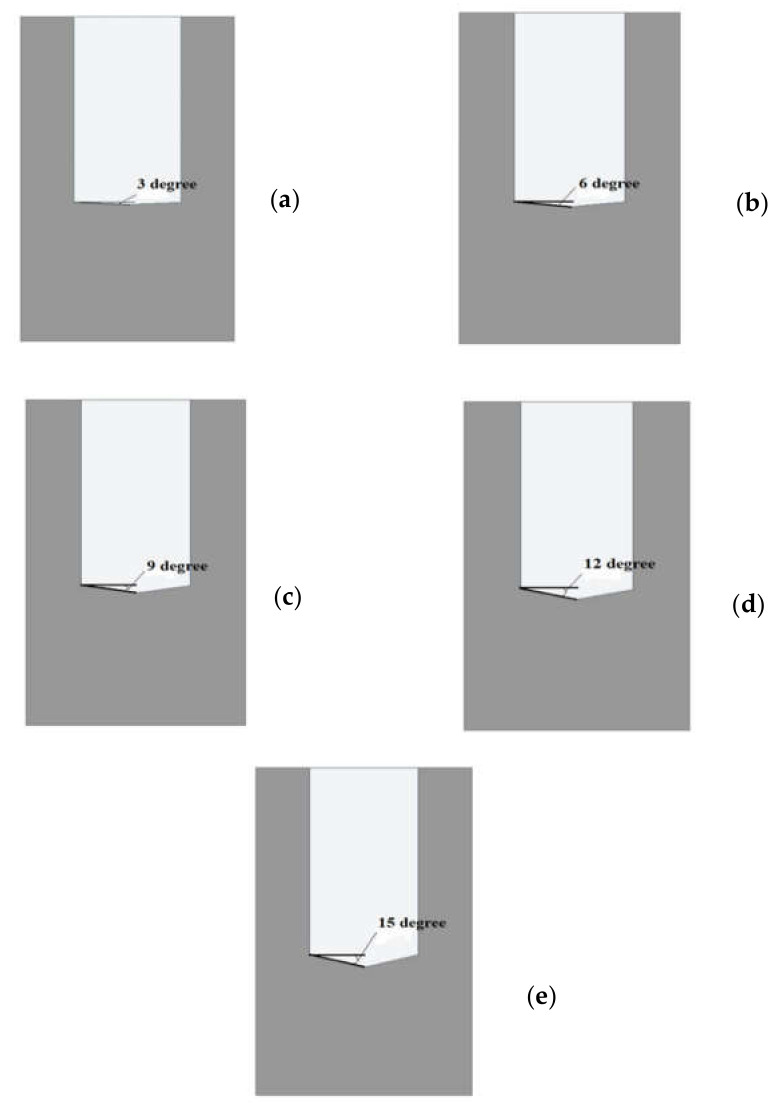
Two sided wedge shape in microchannel (cross-sectional view): angle (**a**) 3°, (**b**) 6°, (**c**) 9°, (**d**) 12°, (**e**) 15°.

The computational domain of microchannel heat sink and fluid was created in the design modular of Ansys workbench. The domain was non-isothermal, single-phase, steady-state, and completely three-dimensional. The computational domain in this work was confined to a symmetrical unit only as the microchannel heat sink was made up of 50 straight channels with a pentagonal cross-section, and that would have required massive computing resources and long simulation periods. The unit consisted of one straight flow microchannel and had dimensions of 0.35 mm total height, 0.2 mm width, and 10 mm length. The flow field area was 0.2 mm tall and 0.1 mm wide, as shown in Figure 1. The base interface of the microchannel and the fluid domain was a two-sided wedge in shape and had a varying angle from 5° to 15°. The two-sided wedge shape in the microchannel at the base is shown in Figure 2. The microchannel bottom plate’s thickness is 0.15 mm.

### 2.2. Conservation Equations

In this sub-section, mathematical governing equations are presented. The following equations were solved for steady-state conditions, laminar flow, and single-phase flow. In this study, water was exploited as heat transfer fluid flowing through a microchannel and considered an incompressible flow.

The mass conservation equation or continuity equation:

∇.(ρ_f_.u) = 0 (1)

The momentum conservation equation:

(u.∇).ρ_f_.u = −∇p + μ_f_.∇^2^ u (2)

The energy equation:

u.∇T = k_f_/(ρ_f_ Cp) ∇^2^T (3)

The energy equation for the microchannel (silicon):

k_s_ ∇^2^. T = 0 (4)

### 2.3. Thermo-Physical Properties of Heat Transfer Fluid and Microchannel Heat Sink

The presented simulations have two following computations domains, i.e., microchannel and heat transfer fluid. Silicon is considered a microchannel material, whereas water is considered a heat transfer fluid. The thermo-physical properties of heat transfer fluid (i.e., water) and microchannel heat sink (silicon) are listed below in Table 1 and Table 2, respectively.

### 2.4. Computational Procedure and Boundary Conditions

**Figure 3:**
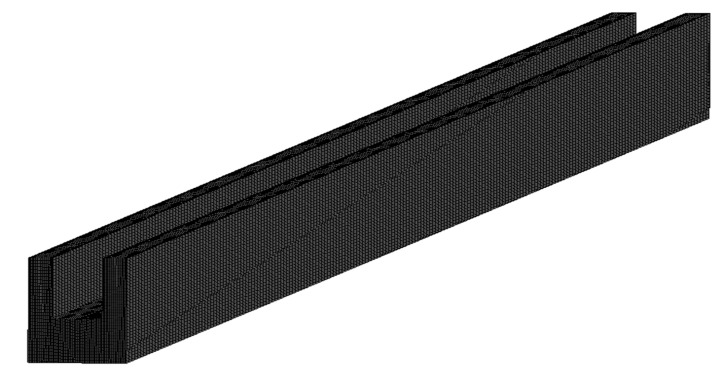
The structured meshing of the microchannel.

Computation domains of single microchannel and fluid were created using the design modular of Ansys Workbench for all simulations. Tetrahedron structured mesh was generated, as shown in Figure 3, which is very fine and can be resolved the boundary layer in the vicinity of walls. Several simulations were carried out to show repeated same results. In order to ensure the mesh independency on the solutions, grid sensitivity tests were carried out. Several solutions were performed by altering the number of nodes and number of elements in the following ranges of 155,737 to 1,299,666 and 143,200 to 1,260,000, respectively. Table 3 shows the effect of the number of nodes and elements on the percentage variation in microchannel temperature. The number of nodes and number of elements increases, and the change in percentage variation in microchannel temperature is very small. Therefore, 1,299,666 nodes and 1,260,000 elements were considered for all simulations further.

The SIMPLE (Semi Implicit Method for Pressure Linked Equations) algorithm was employed for velocity–pressure coupling in the multi-grid solution procedure. An iterative solution was used, with convergence criteria of 1.0 × 10^−9^ for energy and 1.0 × 10^−6^ for velocity component and momentum, which are sufficient to achieve solution convergence. The boundary and initial conditions were given to start the iterative solution. The uniform axial velocity at the inlet was given as per desired Reynolds number in the range of 200–900. The outlet side of the microchannel was susceptible to the pressure outlet. The bottom wall of the microchannel was fixed and exposed to a uniform heat flux of 1.0 × 10^6^ W/m^2^. The side wall of the microchannel was adiabatic, i.e., there was no heat transfer sideways.

### 2.5. Data Acquisition

The numerous data were obtained using the numerical solution in the flow domain and microchannel domain. The data reduction was carried out to present the results in terms of Nusselt number friction factor, pumping power of flow in the microchannel heat sink. The following parameters were evaluated to present the performance:

Reynolds number:

(5) $$Re=\frac{\rho_{f}\mu_{m}\;D_{h}}{\mu_{f}}$$

The hydraulic diameter:

(6) $$D_{h}=\frac{2\;W\;H}{W+H}$$

where *W* and *H* are the width and height of the microchannel, respectively.

The average friction factor:

(7) $$\bar{f}=\frac{\Delta p\;D_{h}}{2\;\rho_{f}\;L\;u_{m}^{2}\;}$$

Average heat transfer coefficient:

(8) $$\bar{h}=\frac{q\;A_{q}}{A_{c}\;\left({\bar{T}}_{c}-{\bar{T}}_{f}\right)}$$

Conjugated area avg temp:

(9) $$\bar{T_{c}}=\frac{\int T\;dA}{\int dA}$$

Mass avg temp coolant:

(10) $$\bar{T_{f}}=\frac{\int T\;\rho_{f}\;dV}{\int \rho_{f}\;dV}$$

The average Nusselt number is given as:

(11) $$\bar{Nu}=\frac{\bar{h\;}D_{h}}{k_{f}}$$

### 2.6. Model Validation

**Figure 4:**
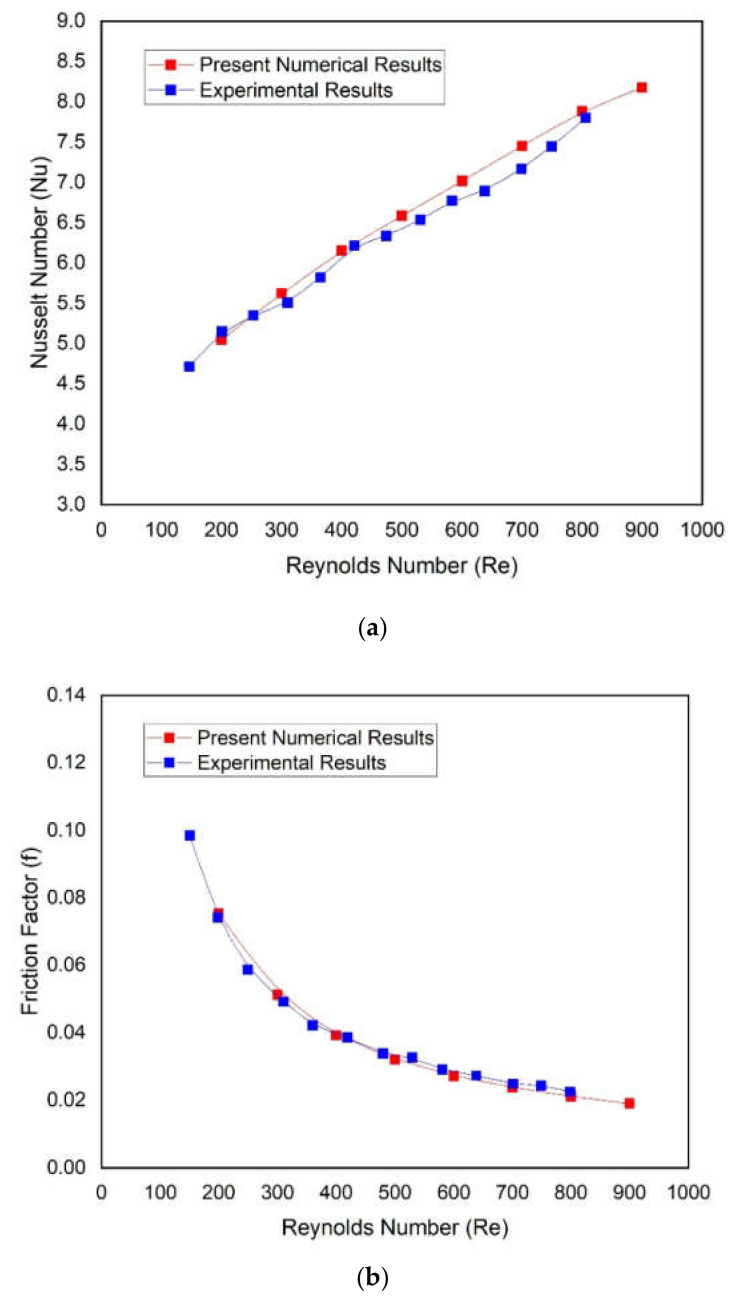
Comparison of present numerical results of (**a**) Nusselt number and (**b**) friction factor, with corresponding experimental results presented by Chai et al. [30].

The single microchannel without a two-sided wedge angle base (conventional microchannel) was simulated, and the results were compared with the well-known experimental study conducted by Chai et al. [30]. Nusselt number and friction factor of conventional microchannel were compared with experimental values of Nusselt number and friction factor of similar microchannel as a function of Reynolds number, as shown in Figure 4. It can be seen from these plots that the numerical values of the Nusselt number and friction factor are in good agreement with the respective experimental values of the Nusselt number and friction factor. It is ensured that the present numerical is reliable, and further simulations can be performed using the same numerical model.

## 3. Result and Discussion

The CFD simulations of the microchannel with a two-sided wedge shape at the base were performed with varying Reynolds numbers from 200 to 900. The base of the microchannel is exposed to a uniform heat flux of 1.0 × 10^6^ W/m^2^. In order to analyze the flow characteristics, the contours of velocity and pressure drop inside the flow domain were presented and discussed. Temperature distribution at the mid plane of fluid and microchannel domain and base of the microchannel was discussed to analyze the heat transfer characteristics in the following sub-sections.

### 3.1. Velocity Distribution

**Figure 5:**
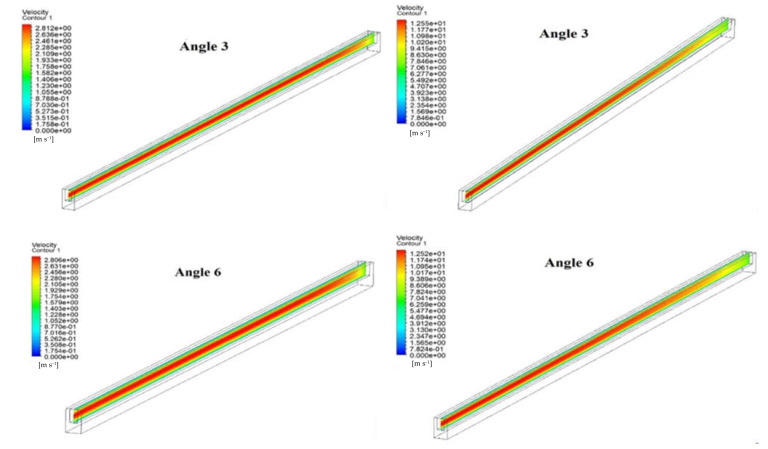

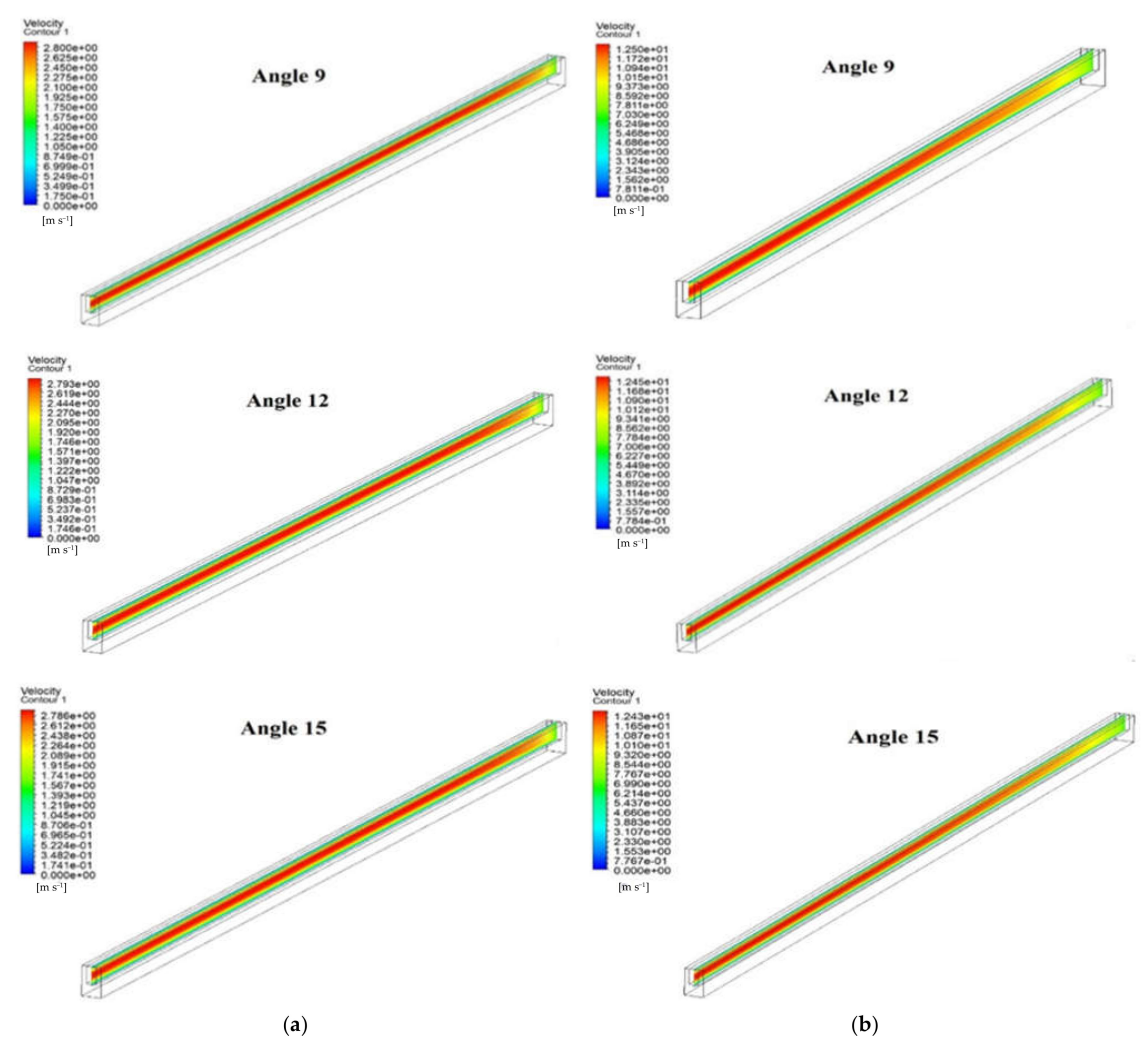
Velocity Contour in microchannel at (**a**) Reynolds Number = 200 and (**b**) Reynolds Number = 900.

The comparison of velocity distribution in the flow field of fluid at different angles and two different Reynolds numbers (200 and 900) are shown in Figure 5. It can clearly be seen that velocity decreases slightly with an increase in angle from 3° to 5° at both Reynolds numbers, i.e., 200 and 900. This happens due to the fact that the surface area of the base of the microchannel increase with the increase in angle. The higher surface area of the base provides a higher heat dissipation to the water. Similarly, velocity decreases with an increase in angle from 3° to 5° in the case of Reynolds number of 900. However, there are significant changes in velocity distribution when the Reynolds number changes from 200 to 900.

### 3.2. Pressure Drop Distribution

**Figure 6:**
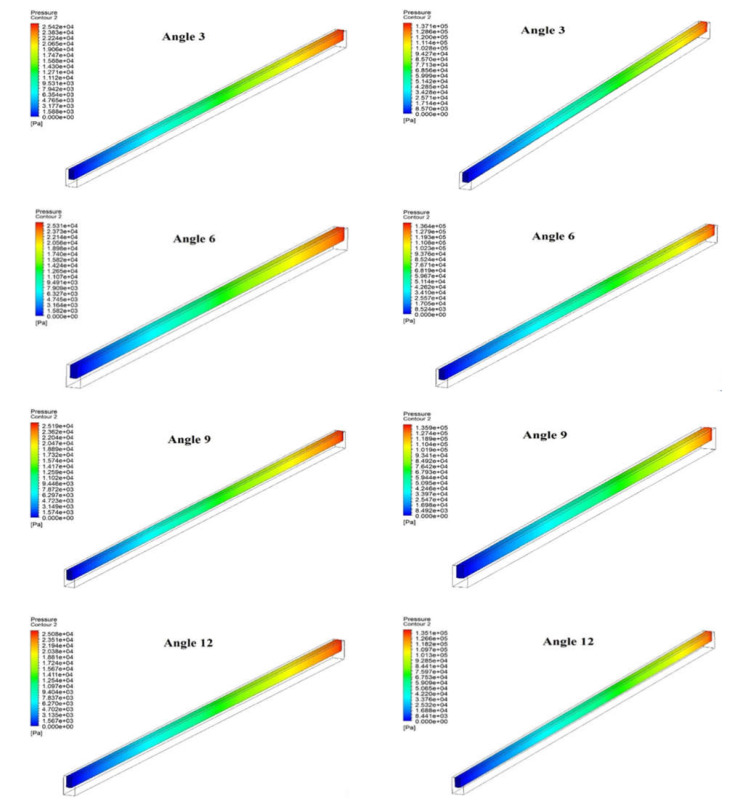

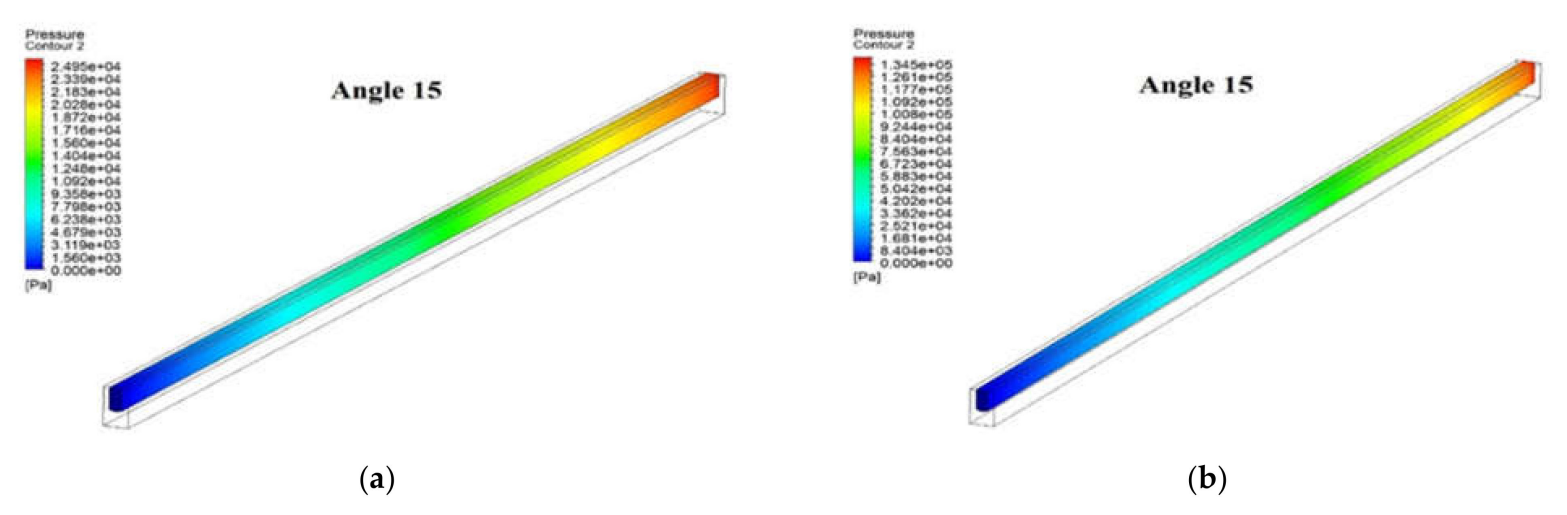
Pressure Contour in microchannel at (**a**) Reynolds Number = 200 and (**b**) Reynolds Number = 900.

Figure 6 depicts the pressure distributions of the fluid domain inside the microchannel when operating with varying Reynolds numbers ranging from 200 to 900. The figure illustrates the pressure drop of the fluid domain in the case of all angles along the heat sink’s microchannel length. At Reynolds number = 200, the maximum pressure drop was found in the case of angle three, and the lowest pressure drop was found at angle 15. A similar trend in pressure drop due to varying angles was found in the case of Reynolds number of 900. However, it can be seen that the difference in pressure drop due to varying angle are not so significant in all cases. Moreover, it can be seen from these contours that the pressure drop for all angles at Reynolds number of 900 is significantly higher than the pressure drop in the case of Reynolds number of 200. It is due to the fact that the viscosity of water restricts the flow at a higher Reynolds number. Therefore, a critical analysis is also required to determine the appropriate base angle that improves heat transmission and lowers pressure drop. 

### 3.3. Temperature Distribution

**Figure 7:**
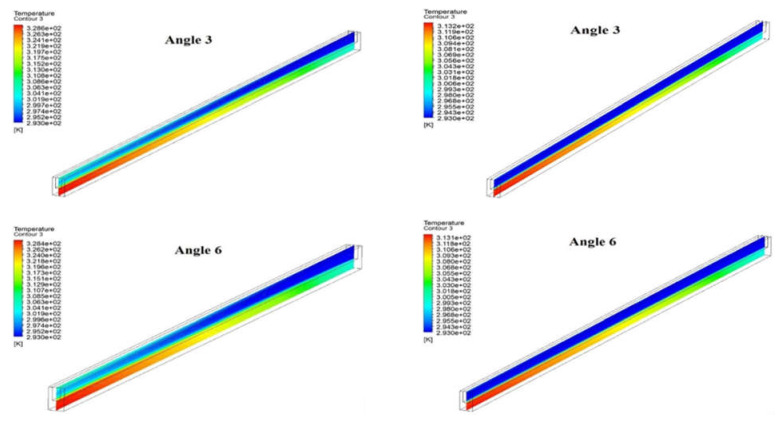

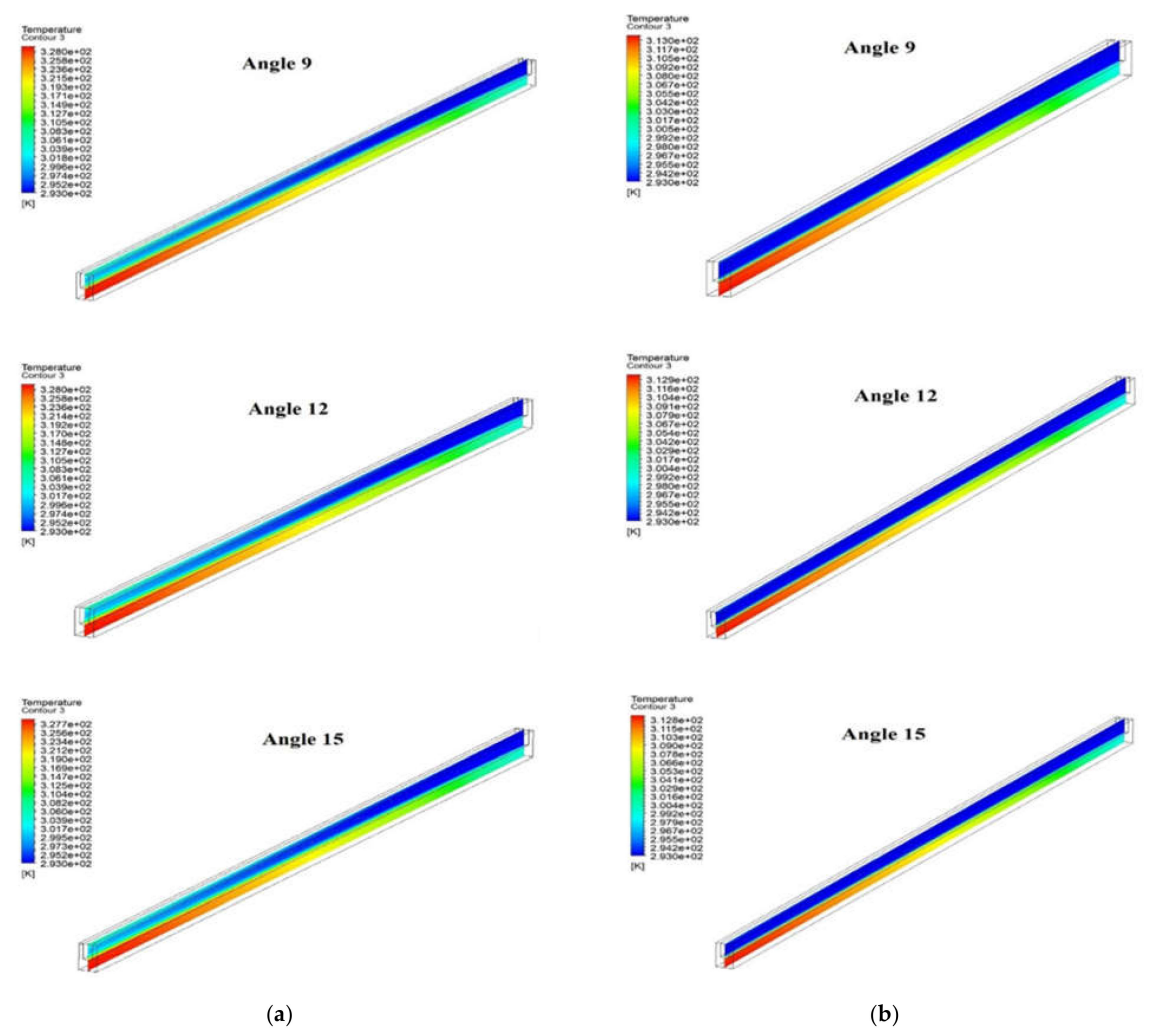
Temperature Contour at mid plane of fluid and microchannel at (**a**) Reynolds Number = 200 and (**b**) Reynolds Number = 900.

Figure 7 shows the temperature distribution at the mid plane of a microchannel heat sink and fluid domain for the different angles at different two Reynolds numbers, i.e., 200 and 900. The bottom wall where the electronic chips are attached shows the maximum temperature. The temperature of coolant flow shows increases in temperature through the flow direction, and the maximum temperature is at the exit of the microchannel. It can be seen that the temperature of fluid increases from inlet to outlet for all angles at both values of the Reynolds number. Moreover, the temperature of the microchannel increase from the fluid inlet to a fluid outlet for all angle at both Reynolds number, which is expected. However, the maximum temperature slightly decreases with an increase in angle from 3° to 15° at both Reynolds numbers. The comparatively lower temperature indicates the heat dissipation rate is higher. Moreover, the maximum temperature decreases significantly when the Reynolds number changes from 200 to 900. It is due to the fact that higher Reynolds numbers extract more and more heat due to suppression of the viscous laminar layer.

**Figure 8:**
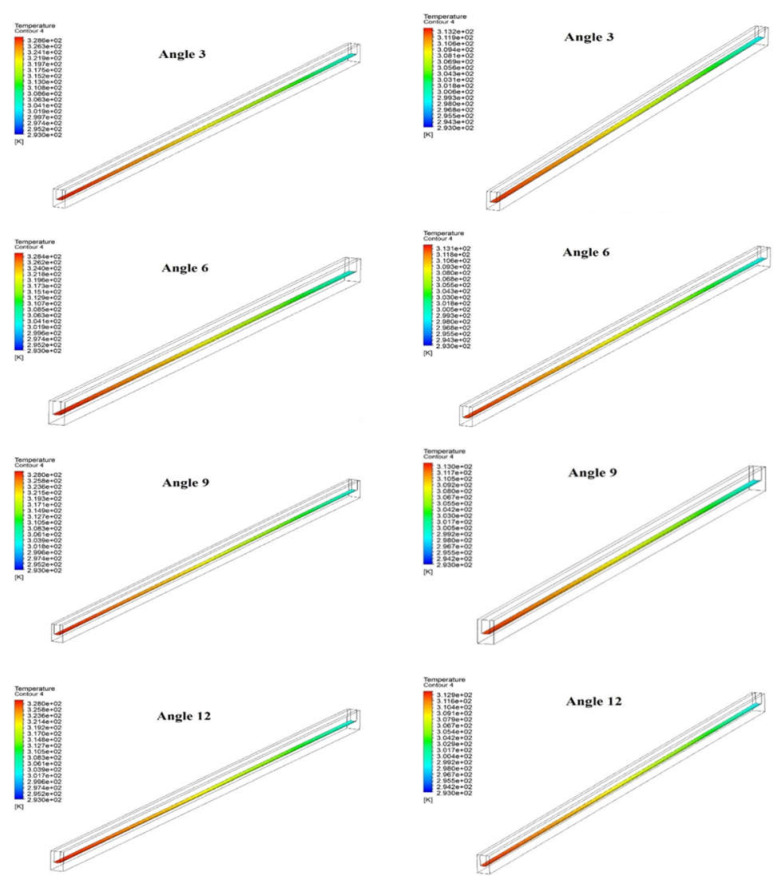

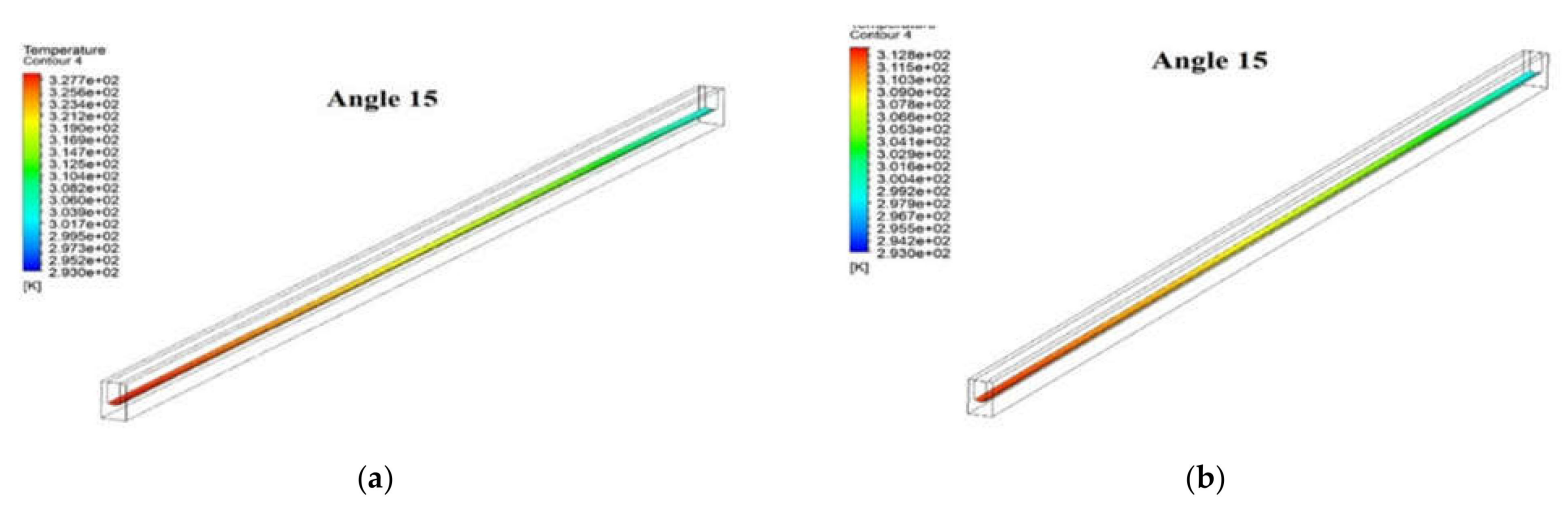
Temperature Contour at base of microchannel at (**a**) Reynolds Number = 200 and (**b**) Reynolds Number = 900.

Figure 8 show the temperature contour at the base of the microchannel for various wedge angle at two values of Reynolds number (i.e., 200 and 900). It can be seen that the temperature distribution over the base of the microchannel is similar, but the maximum temperature slightly decreases with an increase in angles from 3° to 15° at the same Reynolds number, i.e., 200 and 900. This inappreciable change in maximum temperature indicates the appreciable heat dissipation is to be observed at a comparatively higher angle. However, the maximum temperature at the base of the microchannel decrease significantly from 328.6° K to 313.2° K and from 327.7° K to 312.8° K in the case of wedge angle of 3° and 15°, respectively, when the Reynolds number changes from 200 to 900. This significant decrease in maximum temperate indicates that heat dissipation from microchannel to fluid is strongly influenced by changing the Reynolds number.

### 3.4. Performance Analysis

As aforementioned, a rigorous study of the microchannel heat sink is necessary. The heat transfer and fluid flow related to different microchannel heat sink characteristics were presented and analyzed. For a variety of Reynolds numbers, the responses of the heat transfer coefficient, Nusselt number, thermal resistance, coolant pressure drop, and pumping power were obtained to quantitatively characterize the performance of the microchannel heat sink with varying base angles.

**Figure 9:**
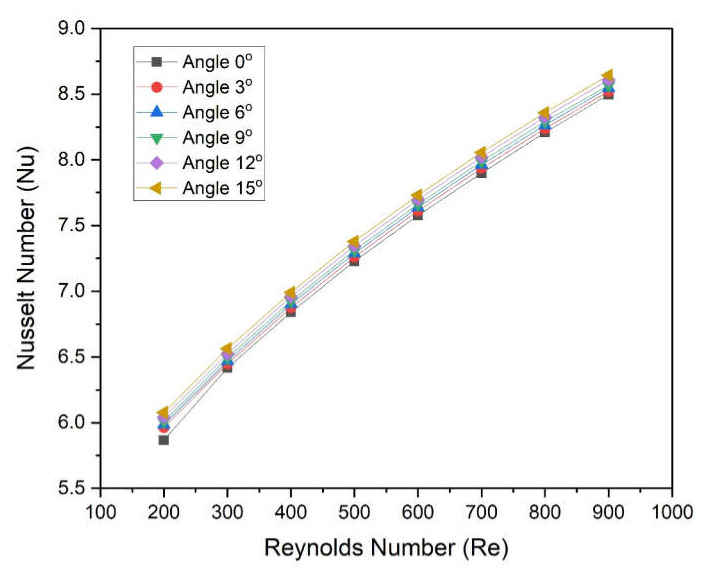
Variation in Nusselt number of microchannel heat sinks with Reynolds numbers for different angles.

Figure 9 shows the variation in the Nusselt number of the microchannel heat sink with Reynolds numbers for different angles. Trends of Nusselt number are found to be continuously increasing concerning Nusselt number for all wedge angles. This trend in Nusselt number is expected. However, higher values of the Nusselt number are found in the case of a 15° angle, and lower values of the Nusselt number are found in the case of a 3° angle for all values of the Reynolds number. The Nusselt number varies in the following ranges of 5.963–8.521, 5.986–8.550, 6.009–8.568, 6.040–8.609, and 6.078–8.644 at 3°, 6°, 9°, 12°, and 15°, respectively. This can be explained using the temperature contour as discussed in the previous sub-section.

**Figure 10:**
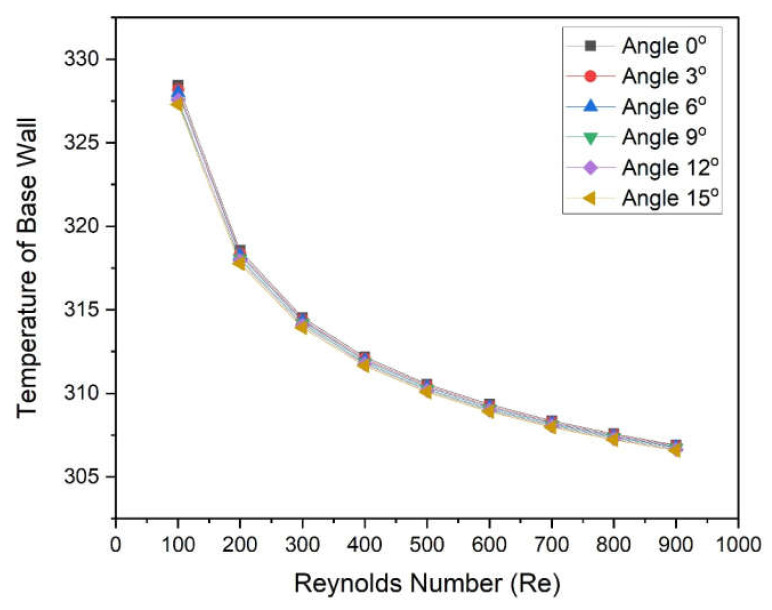
Variation in the temperature of the base wall of microchannel heat sinks with Reynolds numbers for different angles.

In this regard, the temperature of the base of the microchannel with different angles was plotted as a function of the Reynolds number, as shown in Figure 10. It can be observed that base temperature decrease with an increase in Reynolds number for all angles. At a low Reynolds number, temperature decreases a fast rate and becomes asymptotic at higher values of Reynolds number for all Reynolds numbers. For all Reynolds numbers, the lowest base temperatures are found in the case of an angle of 15°, and the highest base temperatures are found in the case of an angle of 3°. The lower temperature at the base of the microchannel in case of a 15° wedge angle contributes to the highest Nusselt number leading to a higher heat dissipation rate.

**Figure 11:**
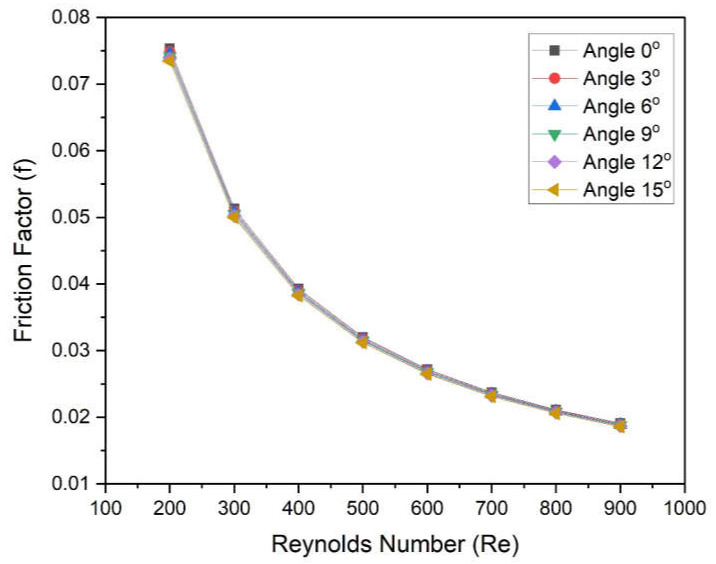
Variation in Friction factor of microchannel heat sinks with Reynolds numbers for different angles.

**Figure 12:**
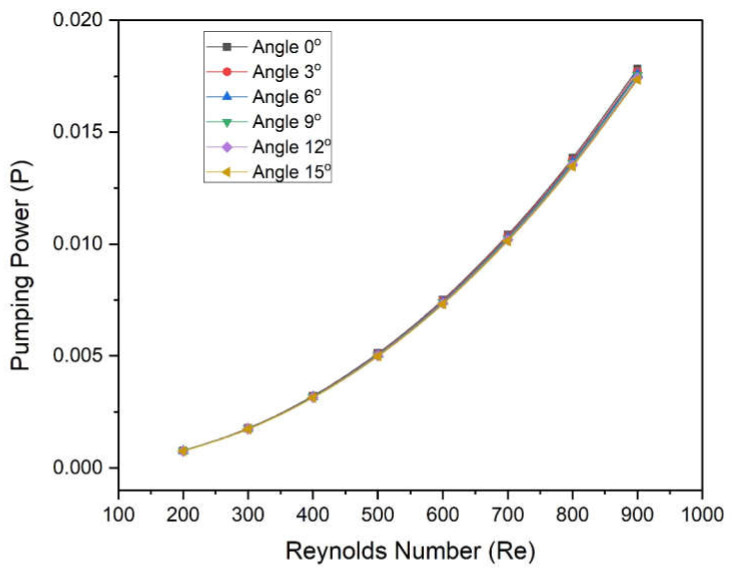
Variation in pumping power of microchannel heat sinks with Reynolds numbers for different angles.

Figure 11 shows the variation in the Friction factor of fluid in a microchannel heat sink with different base angles as a function of Reynolds numbers. The Friction factor decreases continuously with an increased Reynolds number for all values of angles. The rate of decrease in friction factor at a low Reynolds number is found to be higher in comparison to higher values of Reynolds number for all wedge angles. However, the effect of wedge angle on the friction factor is negligible. It indicates the wedge angles do not affect the friction factor. In order to ensure this, Figure 12 was prepared to show the variation in the pumping power of fluid in a microchannel heat sink with Reynolds numbers for different angles. Moreover, the graphs for all different angles are coincident and do not show negligible deviation in pumping power.

In order to distinguish the effect of wedge angle on both heat transfer and friction factor, it is necessary to consider both thermal and hydraulic performance simultaneously, which can show the overall performance. Therefore, a parameter, thermos-hydraulic performance (THPP), *η*, was exploited to show the effect of wedge angle on the overall performance of the microchannel heat sink. If THPP values are greater than unity, then the overall perforce gives fruitful results whatever the individual values of Nusselt number and friction factor. THPP is given below:

(12) η=Nu/Nusf/fs1/3

**Figure 13:**
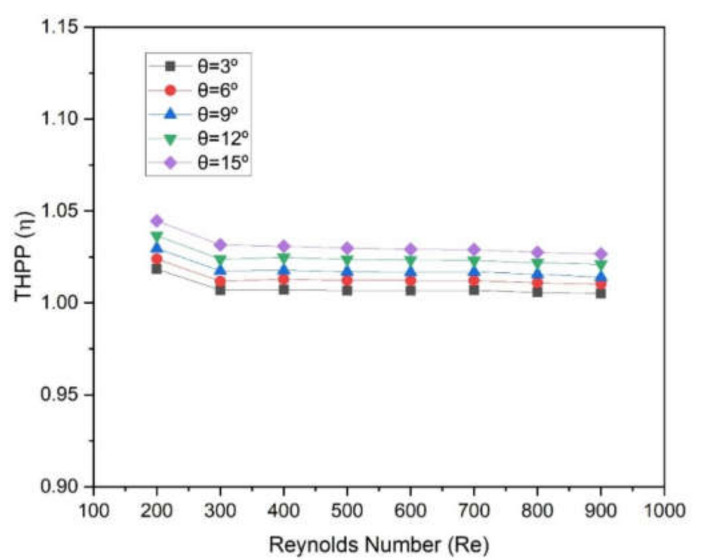
Variation in thermo-hydraulic performance parameter (η) of microchannel heat sinks with Reynolds numbers for different angles.

The values of THPP were evaluated and plotted as a function of the Reynolds number at different wedge angles, as shown in Figure 13 For all angles, the values of THPP decrease slightly with an increase in Reynolds number. These values vary from 1.005 to 1.0185, 1.010 to 1.024, 1.014 to 1.030, 1.021 to 1.037, and 1.027 to 1.045 at a corresponding wedge angle of 3°, 6°, 9°, 12°, and 15°, respectively. It can be seen that all THPP values are greater than 1, which indicates that this design of microchannel has a worth over conventional microchannel. It can be seen that maximum values of THPP were found in the case of a wedge angle of 15°. Therefore, a two-sided wedge angle at the base in a microchannel has an advantage over when there is a flat base in the microchannel.

## 4. Conclusions

The new design of the microchannel was proposed wherein a two-sided wedge angle at the base was incorporated. The five different wedge angles ranging from 3° to 5° were considered. The microchannel with water as heat transfer fluid was simulated using CFD code in Ansys Fluent. On the basis of numerically predicted results following conclusions were drawn:

- Reynolds number significantly affects the Nusselt number as well as the friction factor. The Nusselt number varies in the following ranges of 5.963–8.521, 5.986–8.550, 6.009–8.568, 6.040–8.609, and 6.078–8.644 at 3°, 6°, 9°, 12°, and 15°, respectively;
- The maximum enhancement in Nusselt number is found in the case of an angle of 15°, whereas minimum enactment in friction factor is found in the case of an angle of 3°;
- The wedge angle does not show a significant effect on the friction factor and pumping power. However, the Reynolds number shows a considerable effect on friction factor and pumping power;
- The values of THPP were found to be greater than unity in all cases. Additionally, maximum values were found in the case of a wedge angle of 15°;
- THPP varies from 1.005 to 1.0185, 1.010 to 1.024, 1.014 to 1.030, 1.021 to 1.037, and 1.027 to 1.045 at corresponding wedge angles of 3°, 6°, 9°, 12°, and 15°, respectively.

The numerical study on microchannels presented in this paper is expected to provide a better understanding of microchannel design and development. Since this is a numerical study, it has limitations due to thermo-physical properties of heat transfer fluid and assumptions in boundary conditions. Moreover, an experimental study of such a microchannel is a challenge due to small changes in temperature and temperature measurement of tiny surfaces. In order to solve these issues, thermo-physical properties of the fluid can be altered as desired. In this regard, nanofluids are gaining importance, and numerous nanofluids are being developed so that high-performing and compact microchannels can be developed.

## Figures and Tables

**Table 1:** The thermo-physical property of water at T = 290 K.

Fluid	Density [kg/m^3^]	Dynamic Viscosity μ [Pa·s]	Thermal Conductivity k [W/m·K]	Specific Heat Cp [J/kg·K]
Water	998.2	0.001	0.60	4182

**Table 2:** The thermo-physical properties of heat sink material.

Material	Density ρs [kg/m^3^]	Thermal Conductivity k_s_ [W/m·K]	Specific Heat Cps [J/kg·K]	Young’s Modulus Es [Pa]	Thermal Expansion ℘ [1/K]	Poisson’s Ratio α
Silicon	2329	130	700	170	2.6 × 10^−6^	0.28

**Table 3:** Grid independent test.

S.No.	Nodes	Element	Microchannel Temp T_m_ (°K)	Percentage Variation in T_m_ (%)
1	155,373	143,200	327.79	-----
2	322,203	304,000	327.91	0.037
4	865,104	833,200	327.97	0.018
4	1,299,666	1,260,000	328.01	0.012

## Data Availability

Data available on request.

## Abbreviations

**Nomenclature**	
A_c_	Conjugated area (i.e., the area of the solid-fluid interface) (m^2^)
A_q_	Heated area (i.e., silicon base area) (m^2^)
D_h_	Hydraulic diameter of the microchannel (m)
f	Friction factor
h	Heat transfer coefficient
H	Height of microchannel (m)
Nu	Nusselt number
p	Pressure (Pa)
q	Heat flux that applied to the bottom wall of the micro heat sink (W/m^2^)
Re	Reynolds number
T_c_	Conjugated area average aemperature (K)
T_f_	Mass-average temperature of the coolant in the microchannel (K)
u	Velocity (m/s)
W	Width of microchannel (m)
∆p	Pressure drop
**Greek letters**	
μ_f_	Dynamic viscosity (N.s/m^2^)
η	Thermo-hydraulic performance parameters (THPP)
ρ_f_	Density (kg/m^3^)
**Subscripts**	
f	Fluid (coolant)
h	Hydraulic
s	Solid (silicon) or conventional
